# Expression of *HMGB1*, *TGF-β1*, *BIRC3*, *ADAM17*, *CDKN1A*, and *FTO* in Relation to Left Ventricular Remodeling in Patients Six Months after the First Myocardial Infarction: A Prospective Study

**DOI:** 10.3390/genes15101296

**Published:** 2024-10-02

**Authors:** Jovana Kuveljic, Ana Djordjevic, Ivan Zivotic, Milica Dekleva, Ana Kolakovic, Maja Zivkovic, Aleksandra Stankovic, Tamara Djuric

**Affiliations:** 1 Laboratory for Radiobiology and Molecular Genetics, VINČA Institute of Nuclear Sciences—National Institute of the Republic of Serbia, University of Belgrade, 11000 Belgrade, Serbia; jovana@vin.bg.ac.rs (J.K.); ivanz@vin.bg.ac.rs (I.Z.); anakolakovic@vin.bg.ac.rs (A.K.); majaz@vin.bg.ac.rs (M.Z.); alexas@vin.bg.ac.rs (A.S.); tamariska@vin.bg.ac.rs (T.D.); 2 Faculty of Medicine, University of Belgrade, 11000 Belgrade, Serbia; dekleva.milica@gmail.com

**Keywords:** *HMGB1*, *TGF-β1*, *BIRC3*, *ADAM17*, *CDKN1A*, *FTO*, myocardial infarction, left ventricular remodeling, gene expression, mRNA

## Abstract

**Background:** After myocardial infarction (MI), adverse left ventricular (LV) remodeling may occur. This is followed by LV hypertrophy and eventually heart failure. The remodeling process is complex and goes through multiple phases. The aim of this study was to investigate the expression of *HMGB1*, *TGF-β1*, *BIRC3*, *ADAM17*, *CDKN1A*, and *FTO*, each involved in a specific step of LV remodeling, in association with the change in the echocardiographic parameters of LV structure and function used to assess the LV remodeling process in the peripheral blood mononuclear cells (PBMCs) of patients six months after the first MI. The expression of selected genes was also determined in PBMCs of controls. **Methods:** The study group consisted of 99 MI patients, who were prospectively followed-up for 6 months, and 25 controls. Cardiac parameters, measured via conventional 2D echocardiography, were evaluated at two time points: 3–5 days and 6 months after MI. The mRNA expression six-months-post-MI was detected using TaqMan^®^ technology (Applied Biosystems, Thermo Fisher Scientific, Waltham, MA, USA). **Results:** *HMGB1* mRNA was significantly higher in patients with adverse LV remodeling six-months-post-MI than in patients without adverse LV remodeling (*p* = 0.04). *HMGB1* mRNA was significantly upregulated in patients with dilated LV end-diastolic diameter (LVEDD) (*p* = 0.03); dilated LV end-diastolic volume index (LVEDVi) (*p* = 0.03); severely dilated LV end-systolic volume index (LVESVi) (*p* = 0.006); impaired LV ejection fraction (LVEF) (*p* = 0.01); and LV enlargement (*p* = 0.03). It was also significantly upregulated in PBMCs from patients compared to controls (*p* = 0.005). *TGF-β1* and *BIRC3* mRNA were significantly lower in patients compared to controls (*p* = 0.02 and *p* = 0.05, respectively). **Conclusions:** Our results suggest that *HMGB1* is involved in adverse LV remodeling six-months-post-MI, even on the mRNA level. Further research and validation are needed.

## 1. Introduction

Left ventricular (LV) remodeling plays a critical role in the process of cardiac regeneration after myocardial infarction (MI) [1]. It is a general response to ischemic injury of the myocardium which aims to restore cardiac structure while preserving its function. The process of LV remodeling occurs in three phases: inflammatory, proliferative, and maturation phases [2]. However, the mechanisms responsible for these phases are quite complex and involve multiple pathways. The inflammatory phase begins with hypoxia, which causes ischemic injury, leading to the death of cardiomyocytes and surrounding parenchymal cells. Apoptosis, necrosis, and phagocytosis, together with oxidative stress, activate the inflammatory response. Damage-associated molecular patterns (DAMPs) trigger the secretion and activation of chemokines and cytokines. Various immune cells infiltrate the injured zone and amplify inflammation. During the proliferative phase, fibroblasts and other parenchymal cells transform into a proliferative phenotype, myofibroblasts, which actively secrete new collagen and components of extracellular matrix (ECM). The immune response weakens in this phase, and it should be completely suppressed in the maturation phase [3]. The new ECM ensures scar formation and maturation. In case the inflammation and fibrosis are prolonged, adverse LV remodeling occurs [4]. It is followed by LV hypertrophy (LVH), associated with impairment of LV systolic function and, eventually, heart failure (HF). Excessive collagen and ECM production leads to LV dilatation, which directly affects cardiac structure and function. There is no universal echocardiographic parameter used as a marker for LV remodeling and outcomes after MI; however, LV end-diastolic and end-systolic volumes (LVEDV and LVESV) and diameters and LV ejection fraction (LVEF) are commonly used [5,6]. Nevertheless, it is noteworthy that global longitudinal strain (GLS) emerges as a potential single predictor of LV remodeling and adverse events in patients with acute MI after revascularization at 6–9 months follow-up [7].

Given the complexity of the remodeling process, we have selected several genes, each involved in a specific step of LV remodeling, namely, *HMGB1*, *TGF-β1*, *BIRC3*, *ADAM17*, *CDKN1A*, and *FTO*, to try to portray the elusive moment beyond which the remodeling process from life-saving becomes harmful adverse remodeling. After myocardial infarction, high-mobility group box 1 (HMGB1) has a crucial role—i.e., as a DAMP—in mediating the post-MI inflammatory cascade [8]. It alarms the system and induces the reaction of other molecules which are important for the survival response. One of them, a disintegrin and metalloprotease 17 (ADAM17), is a key regulator of inflammation since it is a membrane-bound shedding protease, also called tumor necrosis factor-α-converting enzyme (TACE), and it is in charge of activating cytokine and chemokine receptors [9]. Successively, HMGB1 promotes transforming growth factor β1 (TGF-β1) signaling and, in this way, mediates fibrosis. Together, HMGB1 and TGF-β1 induce fibroblast transformation and consequential ECM production [10]. *HMGB1*, *ADAM17*, and *TGF-β1* are involved in inflammation and fibrosis, the crucial processes in LV remodeling, while *FTO*, *CDKN1*, and *BIRC3* are responsible for the functioning, proliferation, and apoptosis of the affected cells, especially cardiomyocytes and fibroblasts [11,12,13,14]. Fat-mass-and-obesity-associated gene/protein (FTO), as an RNA demethylase, through selective demethylation of N6 adenosines, affects the contractile function of the cardiomyocytes [11]. Being downregulated after MI [12], *FTO* diminishes cardiomyocyte functioning, while cyclin-dependent kinase inhibitor 1A (*CDKN1A*), also known as p21, plays an inhibitory role in cardiomyocyte proliferation and regeneration [13]. Baculoviral IAP repeat containing 3 (*BIRC3*) encodes a member of the IAP family of proteins, cIAP2, a cellular inhibitor of apoptosis [14]. It protects cardiac fibroblasts from oxidative stress damage, which plays an important role in ischemia–reperfusion injury and heart failure [15]. 

The physiological response of these genes to acute ischemic injury and the early phase of remodeling has been investigated, but changes in their expression in the late phase of LV remodeling are still a field of investigation. In this study, we have followed the change in the echocardiographic parameters of LV structure and function in patients within six months after the first MI. The aim was to analyze the relative expression levels of *HMGB1*, *TGF-β1*, *BIRC3*, *ADAM17*, *CDKN1A*, and *FTO* genes in patients six months after MI and to correlate changes in the LV echocardiographic parameters with these genes’ relative expression in peripheral blood mononuclear cells (PBMCs). In addition, mRNA expression level of the selected genes was determined in the PBMCs of the control group as well.

## 2. Materials and Methods

### 2.1. Study Population

This prospective study consisted of 99 patients who had survived the first MI and 25 healthy controls. All of the participants were unrelated Serbian Caucasians of European descent. Patients were consecutively admitted from December 2011 through September 2013 to the Coronary Care Unit in the Department of Cardiology, University Clinical Center “Zvezdara”, Belgrade, Serbia, with symptoms of the first MI [16] as a consequence of coronary artery disease (CAD). The study group included 82 patients with ST-elevation myocardial infarction (STEMI) who were treated with primary percutaneous coronary intervention (pPCI) within 6 h of symptom onset, and 17 patients with non-ST-elevation myocardial infarction (NSTEMI) who underwent urgent coronary angiography within the first 2 h of hospital admission or within 24 h [17,18]. The exclusion criteria for all patients were as follows: age over 70 years, history of previous MI, HF, cardiomyopathies, heart valve disease, myocarditis, congenital heart disease, atrial fibrillation, previous pacemaker or cardioverter–defibrillator implantation, tumors, chronic inflammatory diseases, autoimmune disease, renal failure, inability to be tested, or deficient echocardiographic imaging. Every patient in the study had stenosis > 70% at the infarct-related coronary artery, including those with single- or multi-vessel CAD, as determined via angiography, which was performed for each patient in compliance with current clinical practice recommendations and standard local procedure. 

Patients’ demographic characteristics, co-morbidities, and risk factors, which included hypertension, diabetes mellitus, hypercholesterolemia, cigarette smoking, alcohol intake, and family history of cardiovascular disease, were recorded at the time of admission. All standard biochemical analyses were performed at the hospital laboratory following routine laboratory procedures, at the time of admission, in hospital, and on the day of discharge. Hypertension was defined as a systolic blood pressure ≥ 140 mmHg, a diastolic blood pressure ≥ 90 mmHg, and/or current treatment with antihypertensive medication. Individuals with a fasting glucose level of ≥7.0 mmol/L or taking insulin or oral hypoglycemic medications were characterized as having type 2 diabetes mellitus (T2DM).

During the six-month follow-up period, patients were prospectively monitored in the same clinic. Cardiac parameters were assessed at baseline (three to five days after MI) and six months after MI using conventional 2D echocardiography.

Control subjects were recruited among the individuals undergoing annual medical check-ups at the Occupational Medical Center, “Vinca” Institute of Nuclear Sciences—National Institute of the Republic of Serbia, Belgrade, Serbia, during the period from February 2011 through December 2013. All of them underwent clinical, ultrasound, and electrocardiogram examinations, and those who did not exhibit any signs of cerebrovascular or cardiovascular disease, chronic inflammatory diseases, T2DM, or renal failure were included in the study. 

The study was approved by the Ethics Committee of the University Clinical Center “Zvezdara”, Belgrade, Serbia (the approval code: NoIRB00003818; approved by Federal-wide Assurance—FWA00006109; approved on 29 June 2011). Each participant provided written informed consent for participation in the study.

### 2.2. Echocardiography

The ultrasound procedure has been previously described [19]. All Doppler echocardiographic examinations were obtained using the second harmonic imaging system Toshiba XG/Artida (Toshiba Medical Systems Corporation, Otawara, Japan) according to the American Society of Echocardiography and the European Association of Cardiovascular Imaging [20]. The examinations were performed at two checkpoints: within three to five days of admission and six months after the MI. 

The following parameters were measured or estimated as previously described [19]: LV end-diastolic diameter (LVEDD); LV end-systolic diameter (LVESD); LV ejection fraction (LVEF); LV end-diastolic volume, indexed to body surface area (BSA) (LVEDVi); and LV end-systolic volume (BSA-indexed) (LVESVi). The estimation of LV mass (BSA-indexed) (LVMi) was obtained by multiplying the volume of the heart by the myocardial density of 1.05 g/mL [20]. Stroke volume (SV) was determined via Simpson’s 2D biplane (4- and 2-chamber views) in accordance with international recommendations [20]. The difference between the value at baseline and six-month follow-up points was used to compute the change (Δ) in cardiac parameters over a six-month period. LVEDD was defined as dilated when LVEDD > 56 mm in men and LVEDD > 51 mm in women [21]. Severely dilated LVESD was defined as LVESD ≥ 46 mm for men and ≥ 42 mm for women [20]. According to the guidelines from the British Society of Echocardiography, the reference intervals for LVEDVi and LVESVi are 30–79 mL/m^2^ and 9–31 mL/m^2^, respectively, in men and 29–70 mL/m^2^ and 8–27 mL/m^2^, respectively, in women. Severely dilated LVESVi was defined as LVESVi >42 mL/m^2^ for men and >37 mL/m^2^ for women. Reference intervals for LVEF are the same for males and females and are ≥55%, while severe systolic dysfunction was defined as LVEF ≤ 35% [22]. LV enlargement (LVE) was defined as a sex-neutral value for LVEDD exceeding 56 mm [20]. LV hypertrophy (LVH) was defined as a LVMi > 115 g/m^2^ for men and >95 g/m^2^ for women [23,24].

Adverse LV remodeling has been conventionally defined as an increase of ≥20% in LVEDV at six months from baseline [4,25]. 

Global longitudinal strains (GLS) were measured from three conventional apical imaging planes; peak systolic strain was defined as the highest deformation at each plane during systole, and the average value was calculated [26].

Development of HF in patients six months after MI was diagnosed according to the European Society of Cardiology Guidelines for the diagnosis and treatment of HF 2012 [27]. Based on the degree of the patient’s symptoms and level of physical activity, HF was classified into the following functional categories: NYHA class I: patients without restrictions on their physical activity; regular physical activity does not cause undue fatigue, palpitation, or dyspnea; NYHA class II: mildly limited physical activity; comfortable at rest; regular physical activity results in exhaustion, palpitation, dyspnea, or chest pain; NYHA class III: significant restriction of physical activity; comfortable at rest; less-than-usual activity causes fatigue, palpitation, dyspnea, or chest pain; NYHA class IV: symptoms of heart failure at rest; any physical activity causes discomfort [28]. Advanced HF was defined as NYHA classes III and IV.

### 2.3. Reverse Transcription Quantitative Real-Time PCR (RT-qPCR)

Whole peripheral blood samples for total RNA extraction were taken from patients six months after MI and from controls. Total RNA extraction from the PBMCs was performed within 30 min of collection using TRIzol^®^ Reagent (Invitrogen, Thermo Fisher Scientific, Waltham, MA, USA) according to the manufacturer’s recommendations. The recombinant Thermo Scientific™ RiboLock RNase Inhibitor effectively protects RNA from degradation at temperatures up to 55 °C (Thermo Fisher Scientific). RNA samples were stored at −80 °C prior to use. BioSpec-nano spectrophotometer was used to evaluate the quantification of RNA (Shimadzu Corporation, Kyoto, Japan). The integrity of the RNA was evaluated by chip electrophoresis using the RNA 6000 Nano Kit (Agilent Technologies, Inc. Headquarters, Santa Clara, CA, USA) on the 2100 Bioanalyzer system (Agilent Technologies). Out of the 99 patients’ samples and 25 controls’ samples collected, 95 and 24, respectively, yielded satisfactory-quality total RNA with an RNA integrity number of 8–9 (ranging from 1 to 10) and were converted to cDNA.

A total of 1 µg of RNA was treated with Thermo Scientific™ DNAse I (Thermo Fisher Scientific), and reverse transcription was conducted using a first-strand cDNA synthesis kit with a Thermo Scientific™ oligo(dT)_18_ primer (Thermo Fisher Scientific) and a Thermo Scientific™ RevertAid Reverse Transcriptase (Thermo Fisher Scientific) in a 20 µL reaction volume in accordance with the manufacturer’s instructions. Real-time PCR was performed in duplicate on the ABI real-time 7500 system (Applied Biosystems, Thermo Fisher Scientific). The detection of *HMGB1*, *TGF-β1*, *BIRC3*, *ADAM17*, *CDKN1A*, and *FTO* expression, as well as that of the reference gene, *Peptidylprolyl isomerase A* (*Cyclophilin A*), was undertaken using the pre-made TaqMan^®^ gene expression assays: Hs00998133_m1, Hs01041915_m1, Hs00355782_m1, Hs01037385_s1, Hs00985031_g1, Hs01057145_m1, and Hs99999904_m1, respectively (Applied Biosystems).

### 2.4. Statistical Methods

An unpaired t-test was used to compare the means of continuous variables that were normally distributed, and the non-parametric Mann–Whitney *U* test was used to compare the medians of continuous variables that were skewed. The values of continuous variables were expressed as mean ± standard deviation (SD). Utilizing Pearson’s Chi-square (χ^2^) test, the categorical variables were compared. A *p*-value of less than 0.05 was considered statistically significant. The relative mRNA levels of the studied genes were standardized against the reference gene *Cyclophilin A* and presented as mean 2^−∆Ct^ values ±SD, where ΔCt represents the difference between the Ct value for the gene of interest and the Ct value for *Cyclophilin A* for each sample. The difference in relative mRNA expression between groups was calculated using a Mann–Whitney *U* test. The changes in the LV echocardiographic parameters within six months following MI (Δ values), analyzed in correlation with the mRNA expression levels of *HMGB1*, *TGF-β1*, *BIRC3*, *ADAM17*, *CDKN1A*, and *FTO*, were tested using Spearman’s rank correlation coefficients (R), and a *p*-value of the correlation <0.05 was considered statistically significant. Multiple linear regression analysis was performed to test if the *HMGB1* mRNA was associated with adverse LV remodeling independently of myocardial infarct size, estimated via peak CK-MB and cardiac Tn, and is presented as a β ± SE coefficient. Statistical analysis was performed using Statistica 8.0 (StatSoft Inc, Tulsa, OK, USA).

## 3. Results

### 3.1. Main Characteristics of Study Population

The general characteristics of the controls and patients with the first MI are shown in Table 1. Patients were older than the controls and had higher triglycerides levels and a higher proportion of men and smokers, while the HDL cholesterol levels were lower than in controls. 

### 3.2. Relative mRNA Expression of HMGB1, TGF-β1, BIRC3, ADAM17, CDKN1A, and FTO in PBMCs from Controls and Patients with MI Six Months after MI

**Figure 1:**
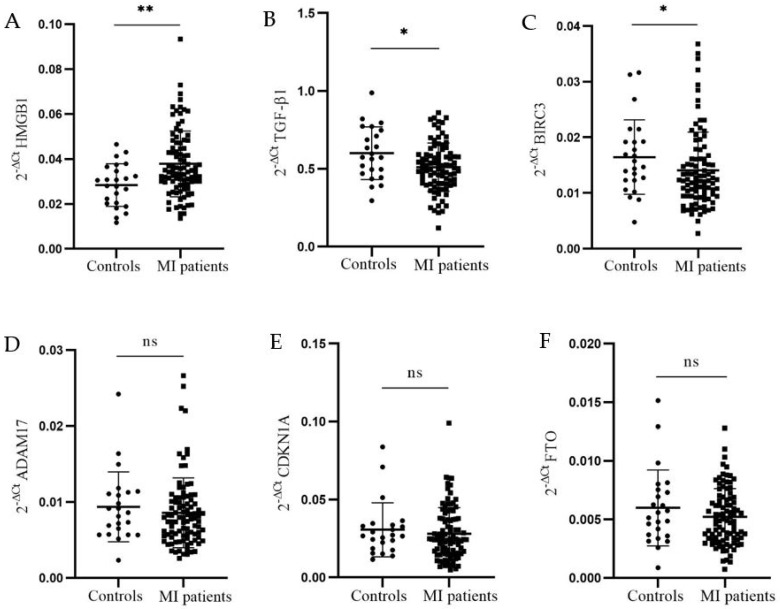
**Relative expression of *HMGB1*, *TGF-β1*, *BIRC3*, *ADAM17*, *CDKN1A*, and *FTO* in controls and MI patients’ PBMCs.** Relative mRNA expression is presented as 2^−∆Ct^ value for each sample. The cDNAs from the PBMCs if controls (N = 24) and patients with MI (N = 95) were used to quantify gene expression. The difference between the Ct values of the reference gene, *Cyclophilin A*, and the gene of interest was used to compute the delta Ct value. Data are presented as 2^−∆Ct^ as mean for both groups (controls—circles; patients—squares) ± SD. The difference of mRNAs relative expression between groups was calculated using the Mann–Whitney *U* test. (**A**) Significant upregulation of *HMGB1* mRNA was detected in PBMCs from patients compared to controls (0.038 ± 0.015 vs. 0.028 ± 0.009, *p* = 0.005); (**B**) *TGF-β1* mRNA was significantly downregulated in PBMCs from patients compared to controls (0.510 ± 0.155 vs. 0.600 ± 0.168, *p* = 0.02); (**C**) *BIRC3* mRNA was significantly downregulated in PBMCs from patients compared to controls (0.014 ± 0.007 vs. 0.016 ± 0.007, *p* = 0.05); (**D**) *ADAM17* mRNA expression was not significantly different between patients and controls (0.009 ± 0.004 vs. 0.009 ± 0.005, *p* = 0.30); (**E**) *CDKN1A* mRNA expression did not differ significantly between patients and controls (0.028 ± 0.017 vs. 0.031 ± 0.017, *p* = 0.40); (**F**) there was no significant difference in *FTO* mRNA expression between patients and controls (0.005 ± 0.002 vs. 0.006 ± 0.003, *p* = 0.39). * *p* < 0.05, ** *p* < 0.01.

Analysis of relative mRNA expression of *HMGB1*, *TGF-β1*, *BIRC3*, *ADAM17*, *CDKN1A*, and *FTO* was performed on PBMCs samples from 95 patients six months after the first MI and 24 healthy controls. Relative mRNA expression of *HMGB1*, *TGF-β1*, *BIRC3*, *ADAM17*, *CDKN1A*, and *FTO* is presented as mean 2^−∆Ct^ values ± SD for each sample. HMGB1 mRNA level was significantly upregulated in PBMCs from MI patients six months after the first MI compared to controls (0.038 ± 0.015 vs. 0.028 ± 0.009, *p* = 0.005) (Figure 1A), while *TGF-β1* and *BIRC3* mRNA levels were significantly lower in patients compared to controls (0.510 ± 0.155 vs. 0.600 ± 0.168, *p* = 0.02 and 0.014 ± 0.007 vs. 0.016 ± 0.007, *p* = 0.05), respectively (Figure 1B,C). The mRNA expression of *ADAM17*, *CDKN1A*, and *FTO* was not significantly different between groups (Figure 1D–F).

### 3.3. Relative mRNA Expression of HMGB1, TGF-β1, BIRC3, ADAM17, CDKN1A, and FTO in Association with Adverse LV Remodeling and Normal Reference Intervals for LV Dimensions, Volumes, and Systolic Function

The general characteristics of patients with the first MI according to the adverse LV remodeling six-months-post-MI are shown in Table 2. Patients with adverse LV remodeling had significantly higher CK_max_, measured 3–5 days after MI, and significantly higher LVEDD, LVEDVi, LVESVi, and LVMi, measured six months after MI than patients without adverse LV remodeling. These patients also had a significantly higher increase in LVEDD, LVEDVi, and LVESVi during the six-month follow-up and a higher proportion of subjects with dilated LVEDD, dilated LVEDVi, and severely dilated LVESVi. 

**Figure 2:**
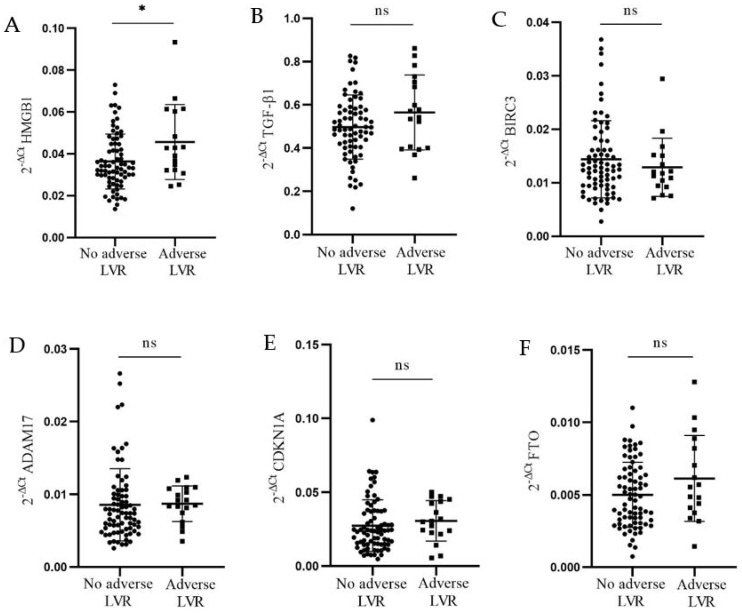
**Relative expression of *HMGB1* and *TGF-β1* in MI patients’ PBMCs, according to the occurrence of adverse LV remodeling six-months-post-MI, based on the >20% increase in LVEDV:** (**A**) significant upregulation of *HMGB1* mRNA was detected in patients’ PBMCs with adverse remodeling (N = 18) compared to patients without adverse remodeling (N = 77) (0.046 ± 0.018 vs. 0.036 ± 0.013, *p* = 0.04); (**B**) *TGF-β1* mRNA expression was not significantly different between patients with adverse remodeling and patients without adverse remodeling (0.565 ± 0.173 vs. 0.497 ± 0.148, *p* = 0.15); (**C**) *BIRC3* mRNA expression did not differ significantly between patients with adverse remodeling and patients without adverse remodeling (0.013 ± 0.005 vs. 0.014 ± 0.007, *p* = 0.48); (**D**) *ADAM17* mRNA expression did not differ significantly between the two patient groups (adverse LVR: 0.009 ± 0.002 vs. without adverse LVR: 0.009 ± 0.005, *p* = 0.15); (**E**) *CDKN1A* mRNA expression was not significantly different between patients with adverse remodeling and patients without adverse remodeling (0.031 ± 0.014 vs. 0.027 ± 0.017, *p* = 0.22); (**F**) *FTO* mRNA expression did not differ significantly between patients with adverse remodeling and patients without adverse remodeling (0.006 ± 0.003 vs. 0.005 ± 0.002, *p* = 0.14). * *p* < 0.05. ns: no significance.

The difference in mRNA expression of targeted genes between patients with (N = 17) and without (N = 75) adverse LV remodeling is presented in Figure 2. It was found that only relative expression of *HMGB1* mRNA in PBMCs from patients with adverse LV remodeling was significantly higher than in patients without adverse LV remodeling six months following MI (0.046 ± 0.018 vs. 0.036 ± 0.013, *p* = 0.04) (Figure 2A). In addition, we conducted multiple linear regression analysis. Which included *HMGB1* mRNA, CK-MB_max_, and Tn_max,_ and found that *HMGB1* mRNA remained significantly associated with adverse LV remodeling, independently of myocardial infarct size (β ± SD = 0.23 ± 0.11, *p* = 0.03).

**Figure 3:**
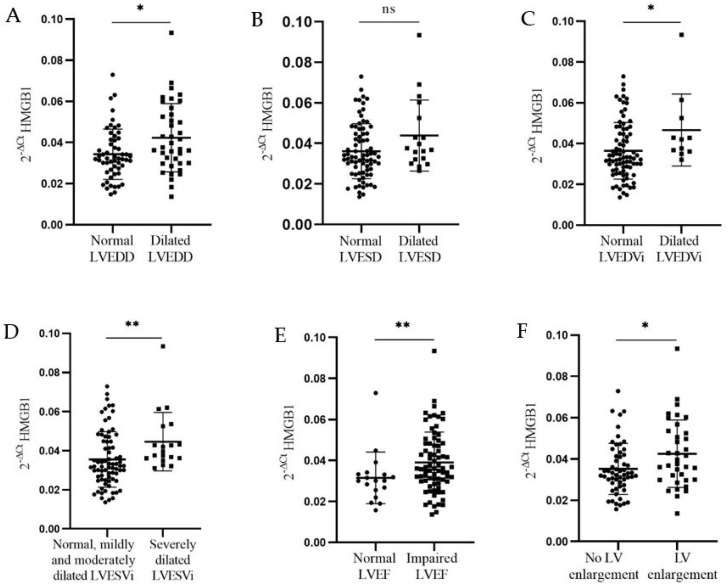
**Relative expression of *HMGB1* in the PBMCs of patients with MI with regard to the reference values of the following echocardiographic parameters: LVEDD, LVESD, LVEDVi, LVESVi, LVEF, and LVE measured six-months-post-MI.** Relative mRNA expression is presented as the 2^−∆Ct^ value for each sample. The delta Ct value was calculated from the difference between the Ct value of the gene of interest and that of *Cyclophilin A*. Data are presented as mean 2^−∆Ct^ ± SD for both groups (normal, reference—circles; dilated/impaired—squares). The Mann–Whitney *U* test was used to calculate the difference in mRNAs’ relative expression between groups. (**A**) Significant upregulation of *HMGB1* mRNA was detected in PBMCs from patients with dilated LVEDD (N = 40) compared to the patients with normal reference LVEDD (N = 55) (0.042 ± 0.017 vs. 0.035 ± 0.012, *p* = 0.03); (**B**) *HMGB1* mRNA expression did not differ significantly between patients with dilated LVESD (N = 19) and patients with normal reference LVESD (N = 76) (0.044 ± 0.018 vs. 0.037 ± 0.013, *p* = 0.11); (**C**) significant upregulation of *HMGB1* mRNA was detected in PBMCs from patients with (mildly, moderately, and severely) dilated LVEDVi (N = 12) compared to the patients with normal reference LVEDVi (N = 83) (0.047 ± 0.018 vs. 0.037 ± 0.014, *p* = 0.03); (**D**) significant upregulation of *HMGB1* mRNA was detected in PBMCs from patients with severely dilated LVESVi (N = 19) compared to the patients with reference and mildly and moderately dilated LVESVi (N = 76) (0.045 ± 0.015 vs. 0.036 ± 0.014, *p* = 0.006); (**E**) significant upregulation of *HMGB1* mRNA was detected in PBMCs from patients with borderline low, impaired, and severely impaired LVEF (N = 76) compared to the patients with normal reference LVEF (N = 19) (0.040 ± 0.014 vs. 0.032 ± 0.013, *p* = 0.01); (**F**) significant upregulation of *HMGB1* mRNA was detected in PBMCs from patients with LV enlargement (N = 37) compared to the patients without LV enlargement (N = 58) (0.043 ± 0.016 vs. 0.035 ± 0.012, *p* = 0.03). * *p* < 0.05, ** *p* < 0.01. ns: no significance.

Relative expression of *HMGB1* was found to be significantly higher in patients with dilated LVEDD compared to those with normal LVEDD (0.042 ± 0.017 vs. 0.035 ± 0.012, *p* = 0.03) (Figure 3A); in patients with mildly to severely dilated LVEDVi compared to those with reference LVEDVi (0.047 ± 0.018 vs. 0.037 ± 0.014, *p* = 0.03) (Figure 3C); in patients with severely dilated LVESVi compared to those with reference LVESVi and mildly and moderately dilated LVESVi (0.045 ± 0.015 vs. 0.036 ± 0.014, *p* = 0.006) (Figure 3D); in patients with borderline low to severely impaired LVEF compared to those with normal LVEF (0.040 ± 0.014 vs. 0.031 ± 0.01, *p* = 0.01) (Figure 3E); and in patients with LV enlargement compared to those without LV enlargement (0.043 ± 0.016 vs. 0.035 ± 0.012, *p* = 0.03) (Figure 3F), all of them at the six-month point following MI.

### 3.4. Relative mRNA Expression of HMGB1, TGF-β1, BIRC3, ADAM17, CDKN1A, and FTO in Correlation with Left Ventricular Structure and Function Echocardiographic Parameters

We have determined if there are correlations between the mRNA of the genes of interest and the change in LV echocardiographic parameters within the six-month follow-up. We found a significant positive correlation of *HMGB1*, *TGF-β1*, and *FTO* mRNA expression levels with ΔLVEDD (R = 0.26, *p* = 0.01; R = 0.21, *p* = 0.05; R = 0.23, *p* = 0.03, respectively). *HMGB1* mRNA level demonstrates a significant positive correlation with ΔLVEDVi, ΔLVESVi, and ΔLVMi (R = 0.25, *p* = 0.02; R = 0.23, *p* = 0.03; R = 0.22, *p* = 0.05, respectively). *CDKN1A* mRNA level demonstrates a significant positive correlation with ΔSV (R = 0.22, *p* = 0.04). *BIRC3* mRNA level demonstrates a significant positive correlation with ΔGLS (R = 0.27, *p* = 0.01) (Table 3).

## 4. Discussion

In this study, we have investigated the relative mRNA expression of six genes implicated in specific steps of LV remodeling: *HMGB1*, *TGF-β1*, *BIRC3*, *ADAM17*, *CDKN1A*, and *FTO*. We investigated whether the mRNA expression of these genes is associated with the occurrence of adverse LV remodeling and whether it correlates with the changes in the echocardiographic parameters serving for the assessment of LV function and structure within six months post-MI in Serbian patients. Their expression was analyzed in the PBMCs of controls as well.

It is known that HMGB1 plays significant role in the pathogenesis of atherosclerosis. During cardiac injury, HMGB1 acts as a DAMP and modulates inflammation. The prolonged immune response leads to excessive post-MI inflammation [29], which is associated with adverse LV remodeling and poor clinical outcomes. In this study, we have found a significantly higher *HMGB1* mRNA level in MI patients compared to controls even six-months post-MI. Very recently, higher serum HMGB1 was found in patients that had acute MI with or without HF compared to controls, whereas the highest level was found in MI with HF [30]. Also, higher levels were found in myocardial and cerebral ischemia compared to controls [31]. A higher serum protein HMGB1 was found in MI patients, even when compared to patients with chronic stable angina [32]. In a rat model of MI, the same authors found an increase in *HMGB1* mRNA on day 3 post-MI, delayed from the expression of two prototypic pro-inflammatory cytokines: TNF-α and IL1-β. *HMGB1* mRNA expression stayed increased for 14 days, even after these cytokines had disappeared. The authors suggested that the active release of *HMGB1* mRNA in this remodeling phase is from the infiltrating inflammatory cells and that it might contribute to the prolonged post-MI inflammatory response leading to adverse remodeling [32]. It seems that the *HMGB1* mRNA levels could have a prolonged presence in circulation and in the heart tissue after the ischemic injury. This could provoke continuous inflammation, which then spreads to the non-infarcted area, leading to the adverse remodeling and HF. Local inflammation following acute MI is also harmful in patients with non-obstructed coronary arteries [33]. In this study, significantly higher levels of *HMGB1* mRNA in the PBMCs of patients six months after MI were found in patients with adverse LV remodeling, severe LV dilatation, impaired LV systolic function, and LV enlargement. In addition, *HMGB1* mRNA was significantly correlated with an increase in left ventricular mass index and LVEDD, both during the six-month follow-up in our study group. All of the above-mentioned cardiac structure and function indicators were previously emphasized as strong prognostic factors for HF [34]. So far, the marked HMGB1 serum elevation after MI has been associated with pump failure, cardiac rupture, and in-hospital cardiac death [32]. It has been correlated with reduced LVEF [30], with infarct size, and was predictive of residual ejection fraction six months after myocardial infarction [35]. Our results on the patients with the first MI who were followed-up for six months, measuring the echocardiographic parameters of LV structure and function and the *HMGB1* mRNA level in PBMCs, contribute knowledge to the previous research that was based on the protein level and animal models. We can suggest that HMGB1, even at the mRNA level, may be a predictor of LV enlargement, severe LV dilatation, impaired systolic function, and adverse LV remodeling after MI, independently of myocardial infarct size, which may eventually lead to heart failure, arrhythmia, and poor clinical outcomes.

TGF-β1 is a multifunctional cytokine that plays a vital role in a plethora of physiological and pathological processes, including vascular remodeling [36]. The data regarding TGF-β1’s pro-inflammatory or anti-inflammatory effect are conflicting, and it seems that TGF-β1 has different and, most of all, cell-specific effects after MI [37,38,39]. A recent single-cell transcriptomic analysis in a murine model found high TGF-β1 expression in the myofibroblasts of healing infarcts, while another myofibroblast subpopulation expressed transcripts for anti-fibrotic proteins, suggesting that the role of TGF-β1 in post-infarct heart remodeling is complex and cell-specific [40]. We found lower levels of *TGF-β1* mRNA in the PBMCs of patients six-months-post-MI compared to the controls free of cardiovascular disease, while the difference in *TGF-β1* mRNA expression between patients with and without adverse LV remodeling was not statistically significant. A recent study conducted on patients with coronary artery ectasia, defined as an enlargement of a coronary artery, the background etiology of which is atherosclerosis, reported lower TGF-β1 plasma levels in patients compared to controls without atherosclerosis [41]. In the endothelial cells and plasma of patients with the obstruction of pulmonary arteries with thrombofibrotic material, the expression of TGF-β1 was increased, but after the removal of thrombotic material from the pulmonary artery, the plasma TGF-β1 levels significantly decreased [42]. These results are in line with the findings of our study; the lower level of TGF-β1 mRNA was found in PBMC of patients six months after MI and after percutaneous coronary intervention, bearing in mind that all the patients had MI as a consequence of ischemic heart disease.

Adverse remodeling after MI could lead to HF, characterized by cardiomyocyte hypertrophy, apoptosis, and necrosis [43]. Even though apoptosis ends wound healing and reduces myofibroblast levels, these cells persist in the infarct scar for years, and *BIRC3* mRNA downregulation in failing LV specimens may increase susceptibility to apoptosis [44,45,46]. In our study, patients had significantly lower levels of *BIRC3* mRNA six-months-post-MI compared to controls, suggesting a greater susceptibility of cells to apoptosis. However, within patients, *BIRC3* mRNA was found to be higher in patients with dilated LV end-systolic diameter, but it did not reach statistical significance. Thus, it may be that in the early phase of remodeling, the resistance of cells to apoptosis plays a role in cardiac tissue repair, but afterwards, it may have a detrimental effect on non-infarcted tissue through hypertrophic scarring and fibrosis. There are very limited scientific data on the role of *BIRC3* in MI or in heart remodeling after MI, and our findings with respect to the *BIRC3* mRNA levels in PBMCs from MI patients followed-up for six months is unique in the literature, so the discussion is rather limited. Nevertheless, we suggest further mechanistic studies on *BIRC3* and validation of its association on larger study groups.

We did not find a significant difference in the *ADAM17* mRNA level between patients six months after MI and controls. In addition, the *ADAM17* mRNA levels were not associated with adverse remodeling or changes in the echocardiographic parameters of LV function or structure six-months-post-MI. Based on the previous literature data, and mainly in the animal models, *ADAM17* mRNA decreases over time after MI [47,48,49,50,51]. Likewise, studies have suggested that ADAM17 is an early marker of cardiac remodeling after MI. They found that *ADAM17* mRNA and protein in the cardiac tissue of the rat model of acute MI were higher at the early stages of remodeling from one week to one month and that the levels decreased to a level that was not significantly different from control rats after 3 months [50]. Since we have analyzed *ADAM17* mRNA six-months-post-MI in the PBMCs of patients, when the acute phase response is finished, we can suggest that ADAM17 is not the informative player for adverse remodeling. Surely, further analysis at different time points and with larger study groups is needed before any conclusion can be drawn for humans.

Previous research has shown that CDKN1A/p21 is associated with an increased risk of atherosclerosis [52] and fibroblast proliferation [53] and may be related to the structural remodeling and progression of MI [54]. In the present study, targeting mRNA expression six-months-post-MI, *CDKN1A* mRNA levels did not differ between controls and patients or between patients with and without adverse LV remodeling. In a study of a mice myocardial ischemia–reperfusion model, the authors induced the inhibition of fibroblast proliferation via p21 overexpression and found that this reduced tissue fibrosis and scar formation [55]. On the other hand, in lincRNA-p21 knockout mice, known as a local transcriptional co-activator of its neighboring gene p21 [56], ventricular wall thickening and deterioration of cardiac function were significantly suppressed [57]. Bearing in mind that CDKN1A is regulated by multiple molecules and affects multiple targets, it would be beneficial for further research to account for the genes that interact with this gene. In addition, there are limited data on the expression of CDKN1A in LV remodelling, so further studies are needed to assess its potential role in LV remodelling after myocardial infarction.

We found no significant difference between the *FTO* mRNA level in PBMCs from controls and patients 6 months after MI, although it was slightly lower in patients. On the other hand, a recent study has shown that the *FTO* mRNA level is upregulated in patients with HF compared to controls [58]. In our study, though on a very small sample size, we also observed significantly higher *FTO* mRNA expression in patients with advanced HF after six months (5 pts; NYHA class III and IV) compared to patients with NYHA class I and II. We also found a significant positive correlation between the *FTO* mRNA level and the enlargement of the LV end-diastolic diameter within a period of six-months post-MI. As the results in the literature are contradictory, further studies in multiple and larger sample groups are needed to assess the potential role of FTO expression in adverse remodeling and HF.

The main limitation of this study is the number of post-MI patients who were prospectively followed. A larger sample group would help to estimate the role of genes with borderline significance in patients with adverse remodeling, such as *TGF-β1*, *ADAM17*, and FTO. This study would benefit from having another follow-up time point, i.e., one year after a myocardial infarction, which would likely include more patients with HF, so that the associations of selected genes could also be investigated in these patients.

## 5. Conclusions

Our results singled out HMGB1 as the most potent gene expressed in the peripheral blood mononuclear cells of post-MI patients in our estimation of adverse LV remodeling, even on the mRNA level. We found its association with LV enlargement, severe LV dilatation, and impaired systolic function six month post-MI, which could subsequently lead to heart failure and poor clinical outcome. However, validation of these results and further research into genes that have been poorly investigated in LV remodeling are required.

## Figures and Tables

**Table 1:** Main characteristics of controls and patients with first acute myocardial infarction.

Variable	Controls, N = 24	MI Patients, N = 95	*p*
Age, years	48.5 ± 8.5	55.2 ± 7.7	0.001 ^§^
Gender, f/m, %	48.0/52.0	22.1/77.9	0.01
BMI, kg/m^2^	26.07 ± 3.28	27.15 ± 3.81	ns ^§^
TC, mmol/L	5.92 ± 0.97	5.46 ± 1.04	ns ^¥^
HDLC, mmol/L	1.52 ± 0.31	1.08 ± 0.27	<0.001 ^§^
LDLC, mmol/L	3.81 ± 0.88	3.52 ± 0.98	ns ^¥^
TG, mmol/L	1.47 ± 0.90	1.86 ± 1.20	0.05 ^§^
T2DM, %	0	34.7	N/A
Hypertension, %	0	48.4	N/A
Current smokers, %	39.1	63.2	0.04

Values are mean ± SD for age, body mass index (BMI), total cholesterol (TC), high-density lipoprotein cholesterol (HDLC), low-density lipoprotein cholesterol (LDLC), and triglycerides (TG). ^¥^—Unpaired *t*-test; ^§^—Mann–Whitney *U* test. Pearson’s Chi-square (χ^2^) test was used for comparison of the categorical variables. *p* < 0.05 was considered statistically significant. T2DM—type 2 diabetes mellitus; ns—non significant; N/A—not applicable.

**Table 2:** Main characteristics of patients with MI with regard to adverse left ventricular remodeling (LVR) six-months-post-MI.

Variable	Patients without Adverse LVR	Patients with Adverse LVR	*p*-Value
	N = 77	N = 18	
Demographic characteristics, laboratory data and risk factors (baseline values)			
Age, years	55.4 ± 7.8	54.3 ± 7.7	0.57
Gender, f/m, %	20.8/79.2	27.8/72.2	0.52
BMI, kg/m^2^	27.04 ± 3.61	26.83 ± 4.29	0.64
TC, mmol/L	5.43 ± 1.04	5.61 ± 1.08	0.52 ^¥^
HDLC, mmol/L	1.07 ± 0.25	1.14 ± 0.33	0.39
LDLC, mmol/L	3.45 ± 0.97	3.79 ± 0.99	0.19 ^¥^
TG, mmol/L	1.94 ± 1.28	1.49 ± 0.70	0.16
T2DM, %	37.7	22.2	0.21
Hypertension, %	49.3	44.4	0.71
Current smokers, %	61.0	72.2	0.38
Glucose, mmol/L	9.67 ± 5.32	8.22 ± 4.52	0.18
CK_max_, U/L	1773.77 ± 1618.62	2955.11 ± 2347.95	**0.05**
CK-MB_max_, U/L	159.29 ± 136.30	247.65 ± 202.03	0.08
Tn_max_, U/L	207.80 ± 842.54	116.85 ± 63.17	0.19
CRP, mg/L	28.30 ± 38.64	22.06 ± 36.38	0.55
HR, beats per minute	75.8 ± 18.1	77.5 ± 12.4	0.29
Systolic blood pressure, mm Hg	129.7 ± 28.2	125.6 ± 24.5	0.77
Diastolic blood pressure, mm Hg	80.5 ± 16.3	76.6 ± 13.1	0.22
Echocardiography (six-month follow-up point)			
LVEDD, mm	54.2 ± 5.8	58.6 ± 8.8	**0.01 ^¥^**
LVESD, mm	39.4 ± 6.5	43.8 ± 9.7	0.06
LVEF, %	46.3 ± 9.0	42.2 ± 9.9	0.1 ^¥^
SV, mL	75.7 ± 13.8	80.7 ± 22.1	0.23 ^¥^
LVEDVi, mL/m^2^	55.2 ± 14.0	69.6 ± 21.2	**0.01**
LVESVi, mL/m^2^	31.8 ± 11.7	42.9 ± 18.3	**0.01**
LVMi, g/m^2^	109.8 ± 26.2	126.0 ± 27.1	**0.02 ^¥^**
∆LVEDD, mm	0.1 ± 4.2	3.7 ± 5.0	**0.002 ^¥^**
∆LVESD, mm	−0.2 ± 4.9	0.9 ± 7.9	0.31
∆LVEF, %	1.5 ± 7.3	−1.1 ± 7.4	0.18 ^¥^
∆LVEDVi, mL	−2.4 ± 10.1	19.9 ± 8.4	**<0.001**
∆LVESVi, mL	−1.1 ± 7.1	13.2 ± 8.9	**<0.001**
∆SV, mL	3.7 ± 16.5	4.6 ± 26.4	0.86 ^¥^
∆LVMi, g/m^2^	−5.2 ± 23.6	12.8 ± 22.1	0.01 ^¥^
Advanced HF, %	4.2	12.5	0.19
LVEF < 55%	77.9	94.4	0.11
Severe systolic dysfunction, %	10.4	22.2	0.17
LVEDD dilated, %	37.7	66.7	**0.02**
LVESD dilated, %	15.6	33.3	0.08
LVEDVi dilated, %	6.5	33.3	**0.001**
LVESVi dilated, %	53.3	72.2	0.14
LVESVi severely dilated, %	16.9	38.9	**0.04**
LVH, %	48.0	72.2	0.06
LVE, %	35.1	55.6	0.11
Post-MI discharge medications (%)			
Aspirin	100	100	1
Clopidogrel	98.7	100	0.63
LMWH	97.4	94.4	0.52
UFH	57.1	77.8	0.11
Nitrates	96.1	88.9	0.22
ACE inhibitors	98.7	94.44	0.26
β-blockers	77.9	77.8	0.99
Diuretics	18.2	16.7	0.88
Statins	98.7	94.4	0.26

Values are mean ± SD for age, BMI, TC, HDLC, LDLC, TG, glucose, creatine kinase (CK)_max_, CK-MB_max_, troponin (Tn)_max_, C-reactive protein (CRP), heart rate (HR), systolic blood pressure, diastolic blood pressure, LV end-diastolic diameter (LVEDD), LV end-systolic diameter (LVESD), LV ejection fraction (LVEF), stroke volume (SV), LV end-diastolic volume index (LVEDVi), LV end-systolic volume index (LVESVi), LV mass index (LVMi), ∆LVEDD, ∆LVESD, ∆LVEF, ∆SV, ∆LVEDVi, ∆LVESVi, and ∆LVMi. Δ—the change of cardiac parameters over a six-month period; ^¥^—unpaired t-test. The Mann–Whitney *U* test was used to compare the values of continuous variables with skewed distribution between controls and patients. Pearson’s Chi-square (χ^2^) test was used for comparison of the categorical variables. *p*-values < 0.05 were considered statistically significant. Advanced HF was defined as NYHA class > 2. Severe systolic dysfunction was defined as LVEF ≤ 35%. LVH—left ventricular hypertrophy; LVE—left ventricular enlargement. LMWH—low-molecular-weight protein; UFH—unfractionated heparin.

**Table 3:** Correlations of *HMGB1*, *TGF-β1*, *BIRC3*, *ADAM17*, *CDKN1A*, and *FTO* relative mRNA expression with the change in left ventricular structure and function echocardiographic parameters within the six-month follow-up after myocardial infarction.

LV Parameter Change (∆)	2^−ΔCt^ HMGB1		2^−ΔCt^ TGFβ		2^−ΔCt^ BIRC3		2^−ΔCt^ ADAM17		2^−ΔCt^ CDKN1A		2^−ΔCt^ FTO	
	R	*p*	R	*p*	R	*p*	R	*p*	R	*p*	R	*p*
∆ LVEDD, mm	0.26	0.01	0.21	0.05	0.08	0.43	0.1	0.32	0.2	0.06	0.23	0.03
∆ LVESD, mm	0.07	0.5	0.09	0.42	0.2	0.06	0.04	0.72	0.06	0.59	0.14	0.19
∆ LVEF, %	−0.13	0.22	−0.13	0.23	−0.16	0.12	0.03	0.73	0.07	0.48	−0.02	0.87
∆ LVEDVi, mL/m^2^	0.25	0.02	0.18	0.09	−0.18	0.08	0.02	0.85	0.06	0.58	0.02	0.84
∆ LVESVi, mL/m^2^	0.23	0.03	0.19	0.07	−0.02	0.84	0.01	0.88	0.02	0.86	0.02	0.81
∆ SV, mL	0.21	0.05	0.2	0.06	−0.01	0.94	0.44	0.67	0.06	0.58	0.04	0.67
∆ LVMi, g/m^2^	0.22	0.05	0.18	0.1	−0.05	0.63	0.19	0.08	0.11	0.31	0.1	0.35
∆ GLS, %	−0.11	0.28	0.001	0.99	0.27	0.01	−0.02	0.84	−0.01	0.92	−0.03	0.74

Values are presented as Spearman’s correlation coefficient (R) and *p* value of the correlation. *p* < 0.05 was considered statistically significant. GLS—global longitudinal strain.

## Data Availability

The original contributions presented in this study are included in the article. Further inquiries can be directed to the corresponding author.
